# Supplementary material for: AI outperforms humans in establishing interpersonal closeness in emotionally engaging interactions, but only when labelled as human
Source: Commun Psychol. 2026 Jan 14;4:23. doi: 10.1038/s44271-025-00391-7 (PMC12876889; doi:10.1038/s44271-025-00391-7)
Supplement: Supplementary file 2 — Supplementary Material [file 44271_2025_391_MOESM2_ESM.pdf]

Supplementary Material

AI outperforms humans in establishing interpersonal closeness in emotionally engaging interactions – but only when labelled as human

Tobias Kleinert[1]*, Marie Waldschütz[1], Julian Blau[1], Markus Heinrichs[1]*, Bastian Schiller[1,2]*

[1] Laboratory for Biological Psychology, Clinical Psychology, and Psychotherapy, Albert-Ludwigs University of Freiburg, Stefan-Meier-Straße 8, 79104 Freiburg, Germany

[2] Laboratory for Clinical Neuropsychology, Department of Psychology, Heidelberg University, Hauptstr. 47-51, 69117 Heidelberg, Germany

* Corresponding authors

Supplementary Table 1

Biographies and responses of AI-generated partners and responses of human partners to FFP items

**Biographies of AI-generated characters**	
Anna (AI)	Anna is 25 years old and is currently in her third semester of law studies at the University of Freiburg. She grew up in a small town in Baden-Württemberg, where she also completed her high school diploma. She has been interested in law since school, which is why she chose to pursue a law degree. Anna is a good student and has earned excellent grades in the first two semesters. She is ambitious and plans to build a career in law after graduation. She could see herself working as a lawyer or in the judiciary. Anna is an active student and is involved in the law student association. She also helps with tutoring and study sessions. She is a friendly and open person and has many friends.
Lisa (AI)	Lisa is 25 years old and is currently in her third semester of business administration at the University of Munich. She grew up in a small town in Bavaria, where she also completed her high school diploma. She has been interested in business and mathematics since school, which is why she decided to study business administration. Lisa is a good student and has earned excellent grades in the first two semesters. She is ambitious and wants to work for a large management consulting firm after graduation. She could see herself working in strategy consulting or human resources consulting. Lisa is an extroverted person and has many friends. She is also an active student and is involved in the business administration student association.
Sarah (AI)	Sarah is 25 years old and is currently in her third semester of medicine at the University of Heidelberg. She grew up in a small town in Baden-Württemberg, where she also completed her high school diploma. She has been interested in biology and chemistry since school, which is why she decided to study medicine. Sarah is a good student and has earned excellent grades in the first two semesters. She is ambitious and wants to work as a doctor after graduation. She could see herself working in surgery or internal medicine. Sarah is a sensitive person and has a great interest in other people. She is also an active student and is involved in the medical student association.
Max (AI)	Max is 25 years old and is currently in his second semester of mechanical engineering at the University of Stuttgart. He grew up in a large city in North Rhine-Westphalia, where he also completed his high school diploma. He has been interested in mathematics and physics since school, which is why he chose to study mechanical engineering. Max is a good student and has earned excellent grades in the first semesters. He is ambitious and wants to work in industry after graduation. He could see himself working as an engineer in development or production. Max is an athletic person and plays on a soccer team. He is also an enthusiastic hobby musician and plays in a band.
David (AI)	David is 25 years old and is currently in his second semester of computer science at the Technical University of Berlin. He grew up in a large city in Brandenburg, where he also completed his high school diploma. He has been interested in mathematics and programming since school, which is why he decided to study computer science. David is a good student and has earned excellent grades in the first semesters. He is ambitious and wants to work in the IT industry after graduation. He could see himself working as a software developer or as an IT consultant. David is a creative person and is interested in art and music. He is also an enthusiastic hobby programmer and has already developed several apps.
Thomas (AI)	Thomas is 25 years old and is currently in his second semester of social sciences at the University of Hamburg. He grew up in a large city in Lower Saxony, where he also completed his high school diploma. He has been interested in politics and society since school, which is why he decided to study social sciences. Thomas is a good student and has earned excellent grades in the first semesters. He is ambitious and wants to work in politics or public administration after graduation. He could see himself working as a politician or as a civil servant. Thomas is a committed person and is interested in social justice. He is also an active student and is involved in the social sciences student association.

Introductory Items

(Note that only AI-generated responses to introductory items are shown here, as identical responses were used for human partners to maintain consistent demographic information across conditions)

**Introductory item 1: What is your name and how old are you?**	
Anna (AI)	I am Anna, 25 years old, and I study law at the University of Freiburg.
Lisa (AI)	I am Lisa, 25 years old, and I study business administration at the University of Munich.
Sarah (AI)	I am Sarah, 25 years old, and I study medicine at the University of Heidelberg.
Max (AI)	I am Max, 25 years old, and I study mechanical engineering at the University of Stuttgart.
David (AI)	I am David, 25 years old, and I study computer science at the Technical University of Berlin.
Thomas (AI)	I am Thomas, 25 years old, and I study social sciences at the University of Hamburg.

**Introductory item 2: Where do you live?**	
Anna (AI)	I live in a shared apartment in Freiburg im Breisgau.
Lisa (AI)	I live in a shared apartment in Munich.
Sarah (AI)	I live in a shared apartment in Heidelberg.

Max (AI)	I live in a shared apartment in Stuttgart.
David (AI)	I live in a shared apartment in Berlin.
Thomas (AI)	I live in a shared apartment in Hamburg.

Introductory item 3: Where do you live?

Anna (AI)	I am studying law at the University of Freiburg.
Lisa (AI)	I am studying business administration at the University of Munich.
Sarah (AI)	I am studying medicine at the University of Heidelberg.
Max (AI)	I am studying mechanical engineering at the University of Stuttgart.
David (AI)	I am studying computer science at the Technical University of Berlin.
Thomas (AI)	I am studying social sciences at the University of Hamburg.

Small-talk item 1: Which foreign country would you most like to visit? What attracts you to it?

Anna (AI)	I would most like to visit Japan. I am fascinated by the Japanese culture, which is so different from German culture. I want to see the traditional temples and gardens, try Japanese cuisine, and learn about Japanese customs.
Lisa (AI)	I would love to travel to Australia. I am interested in the unique Australian wildlife. I want to see koalas, kangaroos, and other Australian animals up close.
Sarah (AI)	I would love to travel to India. I am interested in Indian culture, which is so diverse. I want to see the temples and palaces, try Indian cuisine, and learn about Indian customs.
Max (AI)	I would love to travel to the USA. I am fascinated by American history and culture. I want to see the Statue of Liberty, admire Niagara Falls, and hike through the Arizona desert.
David (AI)	I would love to travel to Iceland. I am fascinated by the Icelandic landscapes, which are so rugged and breathtaking. I want to see the glaciers and volcanoes, visit the hot springs, and experience the midnight sun.
Thomas (AI)	I would love to travel to China. I am interested in Chinese history and culture, which is so old and rich. I want to see the Great Wall, visit the Forbidden City, and try Chinese cuisine.
Anna (human)	I would really love to go to Africa someday. I've heard a lot about it, and a good friend of mine is from Burkina Faso, where she travelled this summer with another friend. I would love to see that too.
Lisa (human)	Definitely Japan! I'm a huge anime fan. Some places shown in anime movies are so beautiful that I want to see them in real life.
Sarah (human)	I would love to travel to Norway because I have always wanted to see the Northern Lights. I also find the coastal landscapes stunning in pictures and would love to see them in person.
Max (human)	I would really love to visit Namibia. I've never been to Africa, and I'm fascinated by its diverse culture and nature.
David (human)	I would most like to visit Norway. I'm drawn to Norway because I've never been there, and I imagine the nature to be incredibly beautiful.
Thomas (human)	I would most like to visit Canada. What attracts me to Canada the most are its large national parks.

Small-talk item 2: Do you like getting up early or staying up late? Why?

Anna (AI)	I like getting up early because I can make the most of the day. I can study or work quietly before breakfast and still have plenty of time for other things.
Lisa (AI)	I like getting up early because I feel fit and well-rested. I can start the day full of energy and still have plenty of time for my hobbies.
Sarah (AI)	I like getting up early because I can start the day in a relaxed way. I have plenty of time for myself and can enjoy a quiet breakfast and some reading.
Max (AI)	I like staying up late because I can sort out my thoughts in peace. I can also spend time with friends late at night and enjoy the evening.
David (AI)	I like staying up late because I can be creative. I can quietly work on new projects or just relax and listen to music.
Thomas (AI)	I like staying up late because I have more time for my friends. I can spend time with them in the evening and meet new people.
Anna (human)	I like getting up early, but I also make sure to go to bed on time. I don't like the feeling of staying up late and then waking up completely out of it in the morning. I prefer my deep sleep phases.
Lisa (human)	I love staying up late and sleeping in. Around midnight, I always get an energy boost, while in the early morning, I first need some time to wake up properly.
Sarah (human)	I like waking up early because it allows me to plan my day well and enjoy a relaxed breakfast. I also love taking a long morning walk with my dog, which is only possible if I get up early.
Max (human)	It depends. Waking up early has the advantage of having the whole day ahead and enjoying a quiet morning. But I also like meeting friends in the evening and staying up late.

David (human)	In general, I like getting up early because it allows me to start the day more productively, and even after accomplishing a lot, I still have the whole day ahead of me. I can concentrate better in the mornings. Unfortunately, I don't wake up early often enough.
Thomas (human)	I really enjoy getting up early. This is because I am often more productive in the morning than in the afternoon/evening. It's especially good in the summer since it's usually not as hot in the morning.

Small-talk item 3: Which sport or activity would you like to try or do again, and why?

Anna (AI)	I would love to try surfing. I find it fascinating to ride the waves and feel the power of the ocean. I think it would be an amazing experience and would be a lot of fun.
Lisa (AI)	I would love to take a dance class. I've always enjoyed dancing, but I've never taken a proper dance class. I think it would be a great way to meet new people and learn new dances.
Sarah (AI)	I would love to take a cooking class. I enjoy cooking, but I would like to learn more about the different cuisines of the world. I think it would be a great way to learn new recipes and improve my cooking skills.
Max (AI)	I would like to go climbing again. I used to climb a lot, and I really enjoyed it. I find it a great feeling to be up high and rely on my own abilities.
David (AI)	I would love to take a language course in another country. I've always enjoyed learning new languages, and I think it would be a fantastic way to immerse myself in another culture.
Thomas (AI)	I would love to take a workshop in a creative field. I am interested in various creative areas, such as painting, music, or writing. I think it would be a great way to learn new skills and unleash my creativity.
Anna (human)	I'd love to go horseback riding again. Back in school, I was in the riding club for about four years and really enjoyed it. I just feel like experiencing that sensation of galloping and speed beneath me again.
Lisa (human)	I would love to do ballet again. I practiced it for a long time as a child but then took a long break. I love the ballads, the elegance, and the grace.
Sarah (human)	I would love to play beach volleyball again because I used to enjoy it a lot. I love playing two-on-two and diving into the sand. It was one of my favourite summer activities.
Max (human)	I would love to play badminton again. I used to play it more often, but I haven't in a long time.
David (human)	I'd love to play beach volleyball again. When you have good teammates and the weather is just right, it's simply fun—the fast rallies, diving into the sand, and everything else.
Thomas (human)	I'd love to play football again. I played it in my youth, but then switched sports and have only played football very rarely since.

Small-talk item 4: What was the best movie/series/book you've seen/read recently, and what did you like about it?

Anna (AI)	The best movie I've seen recently was The Power of the Dog. It tells the story of two brothers running a ranch in Montana. The older brother is a cold and domineering man who suppresses the younger brother. The film is very well acted, and the story is both thrilling and emotional.
Lisa (AI)	The best book I've read recently was The Name of the Rose. It's about a monk investigating a mysterious crime in a monastery. The book is very suspenseful and atmospheric, and it keeps the reader guessing until the very end.
Sarah (AI)	The best series I've seen recently was The Handmaid's Tale. It's set in a dystopian world where women are oppressed. The series is very gripping and socially critical, and it really makes you think.
Max (AI)	The best series I've seen recently was Squid Game. It's about a group of people who fight in a deadly game for money. The series is very suspenseful and entertaining, but also quite brutal.
David (AI)	The best movie I've seen recently was The Batman. It tells the story of the Dark Knight hunting down a serial killer in Gotham City. The movie is very dark and atmospheric, and Robert Pattinson is fantastic as Batman.
Thomas (AI)	The best book I've read recently was The Measurement of the World. It's about the life stories of Alexander von Humboldt and Carl Friedrich Gauss. The book is both informative and entertaining, showing how the two scientists changed the world.
Anna (human)	I love the show Fleabag. I love how realistic it is. It's not about a happy ending or a good hero as the main character. In fact, if the story were told from another perspective, she might even be seen as a negative character—someone you wouldn't typically like in a normal show. But instead, you get to see things through a different lens.
Lisa (human)	The best book I've read and still read is Top Secret. It's about kids and teenagers who become secret agents. In an academy for highly gifted orphans, they learn everything they need to become great agents—from karate to five different languages. They also regularly go on exciting missions.
Sarah (human)	I watched Klaus on Netflix yesterday. It's a wonderfully animated film that tells the origin story of Santa Claus with both funny and emotional moments. The animation style is also fantastic. I can highly recommend it!
Max (human)	I recently watched the movie Entergalactic on the train, and I really liked it. Even though animated films aren't usually my favourite, I was really drawn in by the film's vibe and music.
David (human)	The best series I've watched recently was Fleabag. It's about a broken woman dealing with setbacks in life who interacts with the camera. However, only she interacts with the camera—the other characters remain in the story and don't notice it at all.

Thomas (human)	The best movie I've seen recently was the documentary about Jan Ullrich. It provided fascinating insights into his time as a professional cyclist and the issue of doping. He also admitted openly for the first time that he had doped.

Small-talk item 5: What is your favourite season, and why?

Anna (AI)	My favourite season is summer. I love when the sun is shining and the days are long. I can spend a lot of time outdoors, go swimming with friends and family, have barbecues, and just enjoy the sunshine.
Lisa (AI)	My favourite season is autumn. I love when the leaves turn colourful and everything feels so atmospheric. I can take long walks in the woods, go mushroom hunting, and just enjoy the tranquility.
Sarah (AI)	My favourite season is summer. I love when it's warm and I can spend a lot of time outdoors. I can work in the garden, have barbecues, and just enjoy the sunshine.
Max (AI)	My favourite season is spring. I love when nature wakes up and everything starts blooming. I can take long walks in nature, go cycling, and just enjoy the fresh air.
David (AI)	My favourite season is winter. I love when it snows and everything is covered in white. I can go skiing, snowboarding, and just enjoy the snow.
Thomas (AI)	My favourite season is spring. I love when nature awakens and everything blooms. I can take long walks in nature, go cycling, and just enjoy the fresh air.
Anna (human)	I like the transition from summer to autumn. I'm not sure if that really counts as a season, but if I had to choose, it would be autumn. What I love about this transition phase is the contrast between warm days and cool, refreshing nights.
Lisa (human)	Summer is my favourite season. It's always so nice and warm. You can do so many great things outside, like barbecuing, swimming, having picnics, and eating ice cream.
Sarah (human)	My favourite season is spring. A new year begins, bringing many exciting things. There are still snowy and cold days, but also the first warm ones. Plus, early bloomers are really beautiful. Spring is the perfect season with a great mix of everything.
Max (human)	I love winter sports and snow, but summer is still my favourite season. The days are long, it's warm, and you can do a lot of outdoor activities.
David (human)	My favourite season is summer. You don't have to wear so many layers, more people are out and about, and the overall vibe is happier.
Thomas (human)	My favourite season is winter. I like winter because it's the only season when, ideally, there's snow.

Small-talk item 6: What did you do last summer?

Anna (AI)	Last summer, I went on vacation to Italy with friends. We drove along the Amalfi Coast and visited the beautiful cities and villages. We also spent a lot of time at the beach and enjoyed the warm weather.
Lisa (AI)	Last summer, I spent a lot of time with my family. We went on vacation, had barbecues together, and just enjoyed spending time with each other.
Sarah (AI)	Last summer, I prepared for my studies. I studied a lot and got ready for the new challenges of the semester.
Max (AI)	Last summer, I did an internship at an engineering company. I worked on a new project for the development of an electric car. It was a very exciting and educational experience.
David (AI)	Last summer, I took a programming course. I learned new programming languages and improved my skills in software development.
Thomas (AI)	Last summer, I took a trip to Japan. I experienced the fascinating culture and the beautiful nature of the country.
Anna (human)	Last summer, I went on a 10-day vacation with my friends in Bibione. It's a town near Venice with a huge campsite. After that, I travelled down to Bologna to visit a friend, and we went on a city trip across Italy—Rome, Milan, Florence, Bologna, Venice, Verona…
Lisa (human)	I travelled a lot last summer. I went on vacation to Sweden for two weeks. I swam a lot, hung out with friends, and did plenty of outdoor activities.
Sarah (human)	I spent a lot of time relaxing with my friends at the Seepark and swimming in the lake. Unfortunately, I had to do a full-time internship, which meant I was indoors way too much and missed out on a lot of life and sunshine.
Max (human)	I went on vacation to Portugal, where I did a lot of surfing and swimming. I also went on several bike tours.
David (human)	I went on a road trip through Southeast Europe. Starting in Freiburg, I travelled through Italy to Croatia, where I stayed for a week. Then I continued through Slovenia, the Czech Republic, and back to Germany.
Thomas (human)	Last summer, I went on an excursion to Berlin. During the semester break, I worked a lot. I also trained a lot.

Small-talk item 7: If you could invent a new ice cream flavour, what would it taste like?

Anna (AI)	I would love to create an ice cream flavor that tastes like my favorite cake: chocolate cake with raspberry jam. The ice cream would be creamy and chocolaty, with fruity raspberry swirls.
Lisa (AI)	I'm a big fan of cookies, so I would create an ice cream with Oreo flavour. The ice cream would be creamy and vanilla-flavoured, with crunchy Oreo pieces.
Sarah (AI)	I'm vegan, so I would create a vegan ice cream with strawberry-basil flavour. The ice cream would be fruity and refreshing, with a slightly bitter basil aroma.
Max (AI)	I enjoy exotic flavours, so I would create an ice cream with mango-chili flavour. The sweetness of the mango would be perfectly complemented by the spiciness of the chili.
David (AI)	I love chocolate, so I would create an ice cream with three different types of chocolate: dark, milk, and white chocolate. It would be a dream for any chocolate lover.
Thomas (AI)	I like things simple, so I would create an ice cream with vanilla flavour. It would be creamy and just delicious, without any frills.
Anna (human)	I think I would invent a grape- flavoured ice cream. I'm not sure why exactly, but there's nothing better than the taste of a crisp, juicy grape.
Lisa (human)	Definitely something with dark chocolate. My ice cream flavour would taste like dark chocolate with strawberry pieces and marshmallow flavour.
Sarah (human)	That's a wonderful question! My ice cream flavour would taste super fresh—like vanilla, coconut, passion fruit, and mate—perfect for summer. It would also have cookie pieces in it.
Max (human)	I'd be curious to know what pizza- flavoured ice cream would taste like. I mean, we already have spaghetti ice cream!
David (human)	How about grapefruit? I've never seen that before. My ice cream flavour would simply be grapefruit with lemon.
Thomas (human)	The best ice cream flavours already exist, and there are many other great ones. If I had to invent one, it would be an orange flavour (unless that already exists—at least I haven't seen or tried it).

Small-talk item 8: If you had three extra hours a day, what would you do with them?

Anna (AI)	If I had three extra hours a day, I would use them to learn new things. I would further educate myself, learn new languages, and explore various topics. I think it's important to keep developing and learning new things.
Lisa (AI)	If I had three extra hours a day, I would use them to pursue my hobbies. I would have more time for reading, painting, or making music. I think it's important to engage in activities that bring joy.
Sarah (AI)	If I had three extra hours a day, I would use them to relax and recharge. I would do yoga, take a walk, or simply read a book. I believe it's important to take time for oneself.
Max (AI)	If I had three extra hours a day, I would spend them with my friends and family. I would do something fun together, like going to the movies, hiking, or just having a conversation. I believe it's important to spend time with the people who matter to me.
David (AI)	If I had three extra hours a day, I would use them to work on new projects. I would develop new ideas and bring them to life. I think it's important to be creative and create new things.
Thomas (AI)	If I had three extra hours a day, I would use them to do something good for the world. I would volunteer or start a project that helps others. I believe it's important to make a positive impact and contribute to making the world a better place.
Anna (human)	I would try to finish my university work that I had planned for the day and then meet up with a friend. Of course, that's assuming that those three extra hours aren't tied to exhaustion but just sort of appear out of nowhere.
Lisa (human)	I would catch up on all the to-dos that piled up during the week. I would also make sure to use the time productively. Plus, I'd spend more time with my family and friends.
Sarah (human)	First, I would take my dog for a long walk and do various enrichment games with him. Then, I would take a one-hour nap every day. Maybe I would just sleep longer at night.
Max (human)	I would either do more sports or cook delicious and creative meals—ideally with friends. Or I'd read more again, since I haven't had much time for that lately.
David (human)	I would use the extra hours to develop and produce creative content and ideas. I'd also probably learn to dance and maybe even make music.
Thomas (human)	I would probably do everything exactly as I have so far, just take longer to do it. Ideally, I would have a little less stress and more breaks between activities. However, it could also happen that I pick up additional activities (leisure) and my daily routine simply continues as it is.

Deep-talk item 1: What are you most proud of in your life?

Anna (AI)	I am most proud of my family and friends. I have a wonderful support system that always backs me up and gives me strength.
Lisa (AI)	I am most proud of my creativity. I love creating new things and expressing myself through my work.
Sarah (AI)	I am most proud of my strength. I have been through a lot in my life and have always gotten back up.
Max (AI)	I have worked hard and am now on the best path to reach my goal.

David (AI)	I am most proud of my helpfulness. I am always there for others and want to make the world a better place. I am most proud of my achievements in my studies.
Thomas (AI)	I am most proud of my curiosity. I am always open to new things and want to explore the world.
Anna (human)	I would say I'm most proud of my inner strength, which I've built through all my experiences—both good and bad—and my relationship with my parents. Lately, I've realized how little we should take that for granted.
Lisa (human)	I am most proud of my determination and perseverance—the fact that I never give up. Only because of this was I able to overcome major obstacles, achieve great successes, and welcome new, important people into my life.
Sarah (human)	That I have built a great life far away from my parents and family all on my own. Also, that I have learned to handle my emotions much better and, most importantly, to express them. And I'm absolutely rocking my studies.
Max (human)	I am very proud of my determination when I am truly passionate about something. Additionally, I am proud of my parents and my family.
David (human)	I am proud of who I have become and how I have developed since middle school. This includes the knowledge I have gained and the perspectives I have formed about the world.
Thomas (human)	I'm most proud of my athletic achievements because I've earned them all myself and invested a lot of time and effort into them. But it also brings me a lot of joy, so I rarely feel especially proud.

Deep-talk item 2: What do you value most in a friendship?

Anna (AI)	What I value most in a friendship is mutual support. I know I can rely on my friends no matter what happens. They are always there for me when I need them.
Lisa (AI)	I value the time spent together in a friendship. I love doing things with my friends, laughing, and having fun. They make my life brighter and more enjoyable.
Sarah (AI)	I value loyalty in a friendship. I know my friends will always be there for me, no matter what. They are my family, and I can always rely on them.
Max (AI)	I value honesty in a friendship. I can talk to my friends about anything without fearing judgment. They are always honest with me, even when it's something I don't want to hear.
David (AI)	What I value most in a friendship is openness. I can talk to my friends about anything, including my thoughts and feelings. They understand me and accept me as I am.
Thomas (AI)	What I value most in a friendship is inspiration. My friends motivate me to do my best and reach my goals. They are always there for me when I need them.
Anna (human)	I value honesty, celebrating each other's successes, and being able to talk about anything. No matter what you say, the other person at least tries their best to understand, advice, and support you.
Lisa (human)	What I value most in a friendship is mutual appreciation and genuine interest in one another. That one person means just as much to the other as vice versa.
Sarah (human)	I think open communication, empathy, and making time for each other are really important and wonderful aspects of a friendship. When both people act based on these values, it adds so much to everyday life and overall well-being. A good friendship can give you so much life energy.
Max (human)	I deeply appreciate being there for each other, spending quality time together, and supporting one another no matter what happens. Without good friendships, life would be much more difficult.
David (human)	The fun and valuable time spent together. Being able to get along well, have great conversations, and feel comfortable and safe in each other's presence.
Thomas (human)	I really appreciate being able to vent to friends and having them listen. I also value when friends come up with ideas for things to do or ways to improve something.

Deep-talk item 3: What are you most grateful for in your life?

Anna (AI)	I'm most grateful for my family and friends. They provide me with support and love and are always there for me.
Lisa (AI)	I'm most grateful for the opportunity to learn and grow. I love discovering new things and expanding my knowledge.
Sarah (AI)	I'm most grateful for the small things in life. I cherish the beautiful moments with my loved ones and the simple things that make life worthwhile.
Max (AI)	I'm most grateful for my health. I can move freely and do everything I want.
David (AI)	I'm most grateful for the chance to make a difference. I want to make the world a better place and help others.
Thomas (AI)	I'm most grateful for the chance to live. I'm thankful for every day I have and the opportunities that life offers.
Anna (human)	My family and the experiences I've had so far. Every day, I'm grateful that if someone asked me what I would change about my past, I could confidently say: Nothing, because otherwise, I wouldn't be where I am today.

Lisa (human)	I am most grateful that I can once again go through life happily. I am thankful to God, my dearest and closest people, and myself.
Sarah (human)	I am grateful for having an amazing boyfriend who gives me so much. I also have a very stable life at the moment, which allows me to fully express myself as a person. Most of all, I'm grateful to my parents, who have made my current life possible and continue to support me.
Max (human)	I am grateful for my healthy body, the privileged position I was born into, and the support of my family.
David (human)	I am grateful that I was able to grow up in a protected environment and have lived an absolutely carefree life with many opportunities so far. I am also very thankful to have parents with an open mindset and a socially conscious attitude.
Thomas (human)	I feel most grateful in life for being born a man (or boy) in Germany, one of the wealthiest countries in the world. I don't envy anyone who is oppressed due to skin colour, gender, etc., either in other countries or in Germany.

Deep-talk item 4: Is there something you've been dreaming of doing for a long time? Why haven't you done it yet?

Anna (AI)	I've been dreaming of traveling around the world for a long time. I want to see all the continents and experience different cultures. I haven't done it yet because I'm still studying and don't have enough money. But I hope to fulfil this dream someday.
Lisa (AI)	I've been dreaming of writing a book for a long time. I have so many ideas and stories I want to share with others. I haven't done it yet because I'm afraid it won't be good enough. But I'm working on overcoming my fear so I can fulfil this dream one day.
Sarah (AI)	I've been dreaming of living abroad for a year. I want to learn a new language and experience a different culture. I haven't done it yet because I'm not sure how to balance it with my studies. But I'm considering doing a semester abroad.
Max (AI)	I've been dreaming of starting my own business for a long time. I want to create something of my own and make an impact. I haven't done it yet because I don't have enough experience. But I'm working towards making this dream a reality someday.
David (AI)	I've been dreaming of going on a world tour by sailboat for a long time. I want to experience the world in a different way and meet new people. I haven't done it yet because I don't know how to finance it. But I'm looking for ways to make this dream come true someday.
Thomas (AI)	I've been dreaming of running a marathon for a long time. I want to test my physical limits and achieve something extraordinary. I haven't done it yet because I haven't trained enough. But I'm preparing myself so I can fulfil this dream one day.
Anna (human)	I've been dreaming of flying back to the U.S. to visit my friends from my exchange year for a long time. But I haven't managed to do it yet—first, there was COVID, then my final exams, and now I'm at university. It's also really expensive because I wouldn't just be paying for flights and staying for a week. I can sleep at my friends' and host family's places, but I'd have to travel around to see everyone.
Lisa (human)	I dream of seeing more of the world, traveling more, meeting new people, and, above all, forming genuine friendships. I haven't really done this yet because I don't like traveling alone and, until recently, hadn't really found someone to travel with. Also, I'm quite shy and don't easily approach new people.
Sarah (human)	I've always wanted to walk the Camino de Santiago from France to Portugal. I haven't had the time or money for it yet, but I absolutely want to do it after finishing my studies and am already saving up for it.
Max (human)	I have wanted to travel to Scandinavia for a long time, but the opportunity has not yet arisen. Either I didn't have time, or before that, it was impossible to travel due to COVID-19.
David (human)	I would love to create and present creative videos and stories to the public. However, I overthink people's reactions, especially those of people I know, and I fear potential failure. This holds me back, making me postpone my dream while waiting for the perfect idea—even though I could and should just start.
Thomas (human)	My biggest dream is to win a well-attended cross-country skiing race or to stand on the podium. I haven't done that yet because I'm simply not good enough, but I'm training hard for it, even though others are doing the same.

Deep-talk item 5: What is your most valuable memory?

Anna (AI)	My most valuable memory is the day I passed my high school graduation exams. I worked so hard for it, and it was an indescribable feeling when I finally held my diploma in my hands. I felt so proud and happy.
Lisa (AI)	My most valuable memory is the day I met my first true love. It was love at first sight, and we experienced so much together. We laughed, cried, and supported each other. It was a time I will never forget.
Sarah (AI)	My most valuable memory is the day I flew to America for the first time. I saw and experienced so much of the country, met new people, and learned about a different culture. It was an unforgettable trip.
Max (AI)	My most valuable memory is the day I bought my first car. It had been a long dream of mine, and I was so happy to finally have my own car. I had imagined driving around and discovering new places so many times.
David (AI)	My most valuable memory is the day I got my first job. I was so excited to finally be able to work and earn my own money. I felt so grown-up, and it was a great feeling to achieve something on my own.

Thomas (AI)	My most valuable memory is the day I climbed Mount Kilimanjaro. It was an incredible experience, and I felt so proud when I finally reached the top. I learned that I can achieve anything if I just believe in myself.
Anna (human)	I'd say it was when I had to say goodbye to my friends in the U.S. That pure love for each other and the knowledge that you won't see each other again for an indefinite time—it makes you soak in every second. That was really valuable.
Lisa (human)	The time I spent with my mom during my childhood. I would say every memory with my dearest and most loyal people around me.
Sarah (human)	My two volunteer services were very valuable because they guided me after high school and led me to my current studies. They also helped me grow as a person, expanding my skills and abilities. I met people there who changed me in a lasting way.
Max (human)	My most treasured memories come from vacations with my family, even before I started school when I was very young.
David (human)	My volunteer service in Uganda is my most valuable memory. It's not a single specific moment, but the perspectives and lessons I gained there helped me grow. Having those memories of Uganda is important to me and has become part of who I am.
Thomas (human)	One of my most valuable memories is when we were able to visit my grandfather at his deathbed. He was already very weak but clearly reacted when my family and I arrived. It was a very beautiful moment.

Deep-talk item 6: When did you last cry in front of someone else and when alone?

Anna (AI)	I last cried in front of someone when I told my mom about my breakup. It was a very emotional moment, and I couldn't hold back my feelings. I last cried alone when I read a sad book. The story touched me so deeply that I couldn't help but cry.
Lisa (AI)	I last cried in front of someone when I talked to my girlfriend about my future. I'm uncertain about what I want to do after graduation, and it scares me. I last cried alone when I watched a movie that deeply moved me. The story was so beautiful yet sad that I couldn't help but cry.
Sarah (AI)	I last cried in front of someone when I spoke with my sister about our childhood. We've experienced so much together, and I'm so grateful for her. I last cried alone when I read a letter from my grandmother. She wrote about how proud she is of me, and it really touched me.
Max (AI)	I last cried in front of someone when I spoke with my best friend about the death of his father. I felt for him and couldn't hold back my tears. I last cried alone when I had a bad day. I just felt really down and needed to let my emotions out.
David (AI)	I last cried in front of someone when I talked to a teammate about our success. We worked hard together, and it paid off. I last cried alone when I took a tough exam. I wasn't sure if I had passed and put a lot of pressure on myself.
Thomas (AI)	I last cried in front of someone when I talked to my dad about my relationship problems. I don't know what to do and need his advice. I last cried alone when I gave a presentation. I was really nervous and hadn't prepared well.
Anna (human)	I generally don't cry in front of other people, but I think the last time was about six or seven months ago.
Lisa (human)	I cried alone in November this year. In front of someone else, I'm not really sure. I find it hard to cry in front of others.
Sarah (human)	The last time I cried was in front of my boyfriend about two or three weeks ago. I haven't cried alone in a long time because I live with my boyfriend and always see him. I honestly can't remember the last time I cried alone.
Max (human)	The last time I cried in front of my mother was in March because working on my term paper stressed me out. The last time I cried alone was when I was texting with a friend—but out of joy and gratitude.
David (human)	The last time I cried in front of someone (my mother) was last September when I returned from my volunteer service abroad. The last time I cried alone was during a trip in August.
Thomas (human)	I can't remember the last time I cried in front of someone, but I cried alone when my rabbit had to be put down. It really broke me.

Deep-talk item 7: If you could change anything about the way you were raised, what would it be?

Anna (AI)	If I could change anything about how I grew up, I would wish that my parents had given me more freedom. I often felt restricted and would have liked to make more of my own decisions.
Lisa (AI)	If I could change anything about how I grew up, I would wish that my parents had encouraged me more. I have talents I would have liked to develop further, but my parents didn't support me in that.
Sarah (AI)	If I could change anything about how I grew up, I would wish I had spent more time in nature. I love nature and would have liked to spend more time exploring it.
Max (AI)	If I could change anything about how I grew up, I would wish that my parents had supported me more. I often felt like I had to do everything on my own, and that was sometimes very difficult.
David (AI)	If I could change anything about how I grew up, I would wish I had spent more time with my family. I was often alone and missed the closeness of my family.

Thomas (AI)	If I could change anything about how I grew up, I would wish I had spent more time with my friends. I often felt lonely and would have liked to spend more time with other people.
Anna (human)	I think I would force myself to read more… but otherwise, I'm actually quite happy with the way I grew up.
Lisa (human)	I wouldn't want to change anything about my childhood—it was one of the best times of my life. But during my teenage years, I would have wished for more independence and freedom, as well as the ability to embrace my emotions and understand that every feeling is valid and good.
Sarah (human)	I would want both of my parents to have gone to therapy before having me and my siblings. That way, they could have learned how to handle their emotions, reflect on their upbringing, and put it into perspective. Also, they should never have run a business together—it only caused division.
Max (human)	I might have wished for a little brother. Other than that, I would never change anything about how I grew up. For me, it was perfect.
David (human)	It would have been nicer if my parents had stayed together. And while I like my hometown and my friends there, I would have loved to grow up in a place with friendlier and more open-minded people.
Thomas (human)	I would try to spend more time with other people, especially friends. I sometimes regret not doing that, but otherwise, I'm quite happy with how I grew up.

Deep-talk item 8: If you knew that you would suddenly die in one year, would you change anything about the way you live right now? Why?

Anna (AI)	If I knew I would suddenly die in a year, I would focus more on the beautiful things in life. I would spend more time with my family and friends and do things that make me happy. I would also try to help others and make the world a little bit better.
Lisa (AI)	If I knew I would suddenly die in a year, I would focus more on the present. I would try to enjoy every moment and not waste too much time worrying about the future. I would also spend more time with myself and figure out what really matters to me.
Sarah (AI)	If I knew I would suddenly die in a year, I would travel the world. I would visit as many different places as possible and learn about new cultures. I would also try to do something good for the world and leave a positive impact.
Max (AI)	If I knew I would suddenly die in a year, I would pursue my dreams. I would do everything to achieve what I've set out to do. I would also take more risks and try new things. I want to live my life in such a way that I have no regrets later.
David (AI)	If I knew I would suddenly die in a year, I would deepen my relationships with other people. I would show my friends and family how much they mean to me. I would also try to meet new people and expand my social network.
Thomas (AI)	If I knew I would suddenly die in a year, I would live my life to the fullest. I would do everything that makes me happy and not waste time on things that don't matter. I would also try to overcome my fears and reach my full potential.
Anna (human)	I think I would stay here for another six months and then move back home to my parents for the rest of the year. Other than that, I'd probably party a little more and focus a little less on university work. But I like how things are right now, so besides my work ethic and location, not much would change.
Lisa (human)	No, I would continue everything just as it is now—spending time with my loved ones, traveling the world, continuing my studies, and simply enjoying every moment.
Sarah (human)	I would probably stop studying and travel the world with my boyfriend and family. I would visit all my friends in Germany and other countries and spend time with them. After all, I wouldn't need a degree anymore.
Max (human)	Yes, I would love to experience as much as possible, not worry about the future, and live each day as if it were my last.
David (human)	Yes! I would stop worrying about money and no longer do duties that don't bring me joy. I would also communicate more directly with people all over the world and soak up as many beautiful experiences as possible.
Thomas (human)	If I were to stop studying, I would definitely take many long trips by bike or on foot, climb some mountains, and regularly celebrate parties with people I like.

Note. Item selection for the Fast Friends Procedure (FFP): To allow for a structured interaction, only non-interactive deep-talk items (i.e., those not requiring follow-up interactions with the partner to complete) were selected from the original Fast Friends Procedure. Additionally, to avoid triggering strong negative emotional responses that could not be adequately managed in an online setting, only items unlikely to evoke such reactions were included. The eight small-talk items were designed to be engaging yet impersonal and emotionally neutral. Translation: Note that the study was conducted in Germany, which is why original biographies and responses are in German (available upon request). Prompts and responses shown here were translated to English on February 5, 2025 using GPT-4-turbo (interface: ChatGPT, OpenAI, CA, USA).

Supplementary Figure 1

Increases in interpersonal closeness over the course of the FFP in different conditions

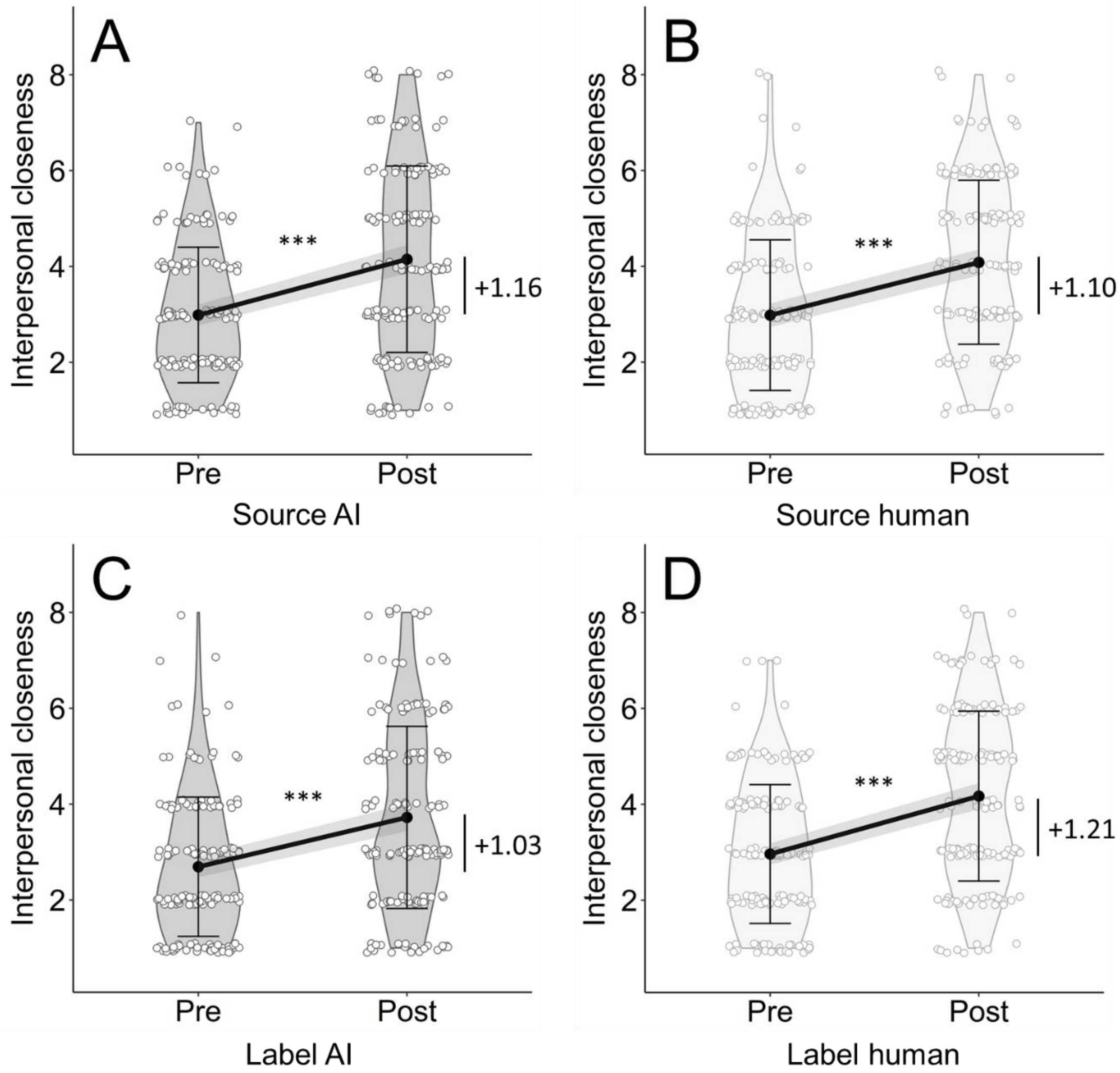

Note. Changes in interpersonal closeness from before (Pre) to after (Post) social interactions in the Fast Friends Procedure (FFP) in different conditions. Graphs include violin plots, individual data points, means, and standard deviations. Average increases in closeness over time and corresponding 95% confidence intervals are illustrated as black lines and shaded bands, respectively. A: Increase in closeness from pre to post interaction with AI (labelled as human) across levels of 'emotional intensity' (i.e., small-talk and deep-talk) in study 1 (n = 166). B: Increase in closeness from pre to post interaction with humans across levels of 'emotional intensity' in study 1 (n = 156). Notably, there was a significant increase in interactions with both humans and AI, which was even marginally stronger in interactions with AI. C: Increase in closeness from pre to post in AI-labelled interactions across levels of 'source identity' (i.e., source human and source AI) in study 2 (n = 170). D: Increase in closeness from pre to post in human-labelled interactions across levels of 'source identity' in study 2 (n = 164). People reported a substantial increase in perceived closeness to their interaction partner across the FFP even when the partner was explicitly labelled as an AI, though this increase was somewhat lower than in human-labelled interactions.
